# Importance of CD8 Tex cell-associated gene signatures in the prognosis and immunology of osteosarcoma

**DOI:** 10.1038/s41598-024-60539-z

**Published:** 2024-04-29

**Authors:** Yining Lu, Nana Cao, Ming Zhao, Guochuan Zhang, Qi Zhang, Ling Wang

**Affiliations:** 1 Department of Orthopedic Research Center, The Third Hospital of Hebei Medical University, Shijiazhuang, Hebei People’s Republic of China; 2 Department of Orthopedic Oncology, The Third Hospital of Hebei Medical University, Shijiazhuang, Hebei People’s Republic of China; 3 Blood Transfusion Department of the Fourth Hospital of Hebei Medical University, Shijiazhuang, Hebei People’s Republic of China

**Keywords:** Osteosarcoma, T cell exhaustion, T cell-related genes, Transcriptomics analysis, Prognostic signature, Tumor microenvironment, Immunotherapy, Bone cancer, Tumour immunology

## Abstract

As a highly aggressive bone malignancy, osteosarcoma poses a significant therapeutic challenge, especially in the setting of metastasis or recurrence. This study aimed to investigate the potential of CD8-Tex cell-associated genes as prognostic biomarkers to reveal the immunogenomic profile of osteosarcoma and guide therapeutic decisions. mRNA expression data and clinical details of osteosarcoma patients were obtained from the TCGA database (TARGET-OS dataset). The GSE21257 dataset (from the GEO database) was used as an external validation set to provide additional information on osteosarcoma specimens. 84 samples from the TARGET-OS dataset were used as the training set, and 53 samples from the GSE21257 dataset served as the external validation cohort. Univariate Cox regression analysis was utilized to identify CD8 Tex cell genes associated with prognosis. The LASSO algorithm was performed for 1000 iterations to select the best subset to form the CD8 Tex cell gene signature (TRS). Final genes were identified using the multivariate Cox regression model of the LASSO algorithm. Risk scores were calculated to categorize patients into high- and low-risk groups, and clinical differences were explored by Kaplan–Meier survival analysis to assess model performance. Prediction maps were constructed to estimate 1-, 3-, and 5 year survival rates for osteosarcoma patients, including risk scores for CD8 Texcell gene markers and clinicopathologic factors. The ssGSEA algorithm was used to assess the differences in immune function between TRS-defined high- and low-risk groups. TME and immune cell infiltration were further assessed using the ESTIMATE and CIBERSORT algorithms. To explore the relationship between immune checkpoint gene expression levels and the two risk-defined groups. A CD8 Tex cell-associated gene signature was extracted from the TISCH database and prognostic markers including two genes were developed. The high-risk group showed lower survival, and model performance was validated by ROC curves and C-index. Predictive plots were constructed to demonstrate survival estimates, combining CD8 Tex cell gene markers and clinical factors. This study provides valuable insights into the molecular and immune characteristics of osteosarcoma and offers potential avenues for advances in therapeutic approaches.

## Introduction

Osteosarcoma (OS) is a significant malignancy affecting bone tissue, particularly prevalent in the adolescent population^1^. This disease is characterized by frequent vascular infiltration, adjacent soft tissue involvement, a notable tendency for local recurrence, and premature distant metastasis^2^. Approximately one-fifth of OS patients develop metastatic lesions, while the remaining patients usually develop subclinical micrometastases. Standard treatment involves chemotherapy and surgical resection^3^. Despite multimodal approaches such as conventional multiagent chemotherapy, surgery, or high-dose chemotherapy combined with stem cell transplantation, patients diagnosed with metastatic or recurrent osteosarcoma still experience poor outcomes, with less than 30% achieving long-term survival^4^. Moreover, due to the young age of onset, the side effects of these treatments can be devastating and persistent. Even patients in remission may face long-term complications, including secondary malignancy, disfigurement (surgery), and psychosocial trauma^5,6^. The complexity and instability of the genome significantly impact treatment outcomes^7^, necessitating the identification of new prognostic genetic markers to predict OS prognosis and guide treatment options. Recently, immunotherapeutic approaches, including over-the-counter cell therapy, vaccination, and immune checkpoint inhibitors, have emerged as potential therapeutic strategies^8^.

While T-cell immunotherapy has demonstrated efficacy against many high-risk malignancies, its effectiveness against osteosarcoma remains largely unexplored. Preclinical studies utilizing immune checkpoint inhibitors (ICIs), antigen-specific chimeric antigen receptors (CARs), or bispecific antibodies (BsAb) have shown the impressive anti-tumor capacity of T cells. However, the immunosuppressive tumor microenvironment (TME) remains a major obstacle^9–12^. Osteosarcoma and TME interact through various environmental signals, such as cytokines, chemokines, and soluble growth factors^13^, hindering immune surveillance while promoting tumor growth and metastasis. This osteosarcoma-specific TME impedes T cell infiltration into the tumor, accelerates immune effector cell exhaustion and inactivation, and disrupts antitumor immunity, creating a significant barrier and potential tumor vulnerability. T cell depletion is a state of T cell dysfunction characterized by poor effector cell function, persistent expression of inhibitory receptors, and a different functional effector cell or memory T cell state than the transcriptional state, which can occur in many chronic infections and cancers^14^. CD8+ T-cell depletion often hampers optimal control of infections and tumors, posing a major obstacle to current anticancer immunotherapies^15–17^. An increased understanding of CD8+Tex and the underlying regulatory mechanisms may open new therapeutic avenues, including strategies related to stimulating and stabilizing effector states during the exhaustion continuum^18–20^.

The primary aim of this study was to explore the potential of CD8-Tex cell-associated genes as biomarkers for assessing risk in osteosarcoma patients. Through a comparison of gene expression patterns between high- and low-risk groups, we analyzed differentially expressed genes (DEGs) and investigated potential molecular mechanisms, regulatory pathways, and immune cell infiltration. The main objective was to elucidate the immunogenomic profile of osteosarcoma and identify survival-associated genes that could serve as valuable clinical biomarkers, guiding treatment planning.

## Result

### Extraction of CD8 Tex cell-associated gene signature

**Figure 1:**
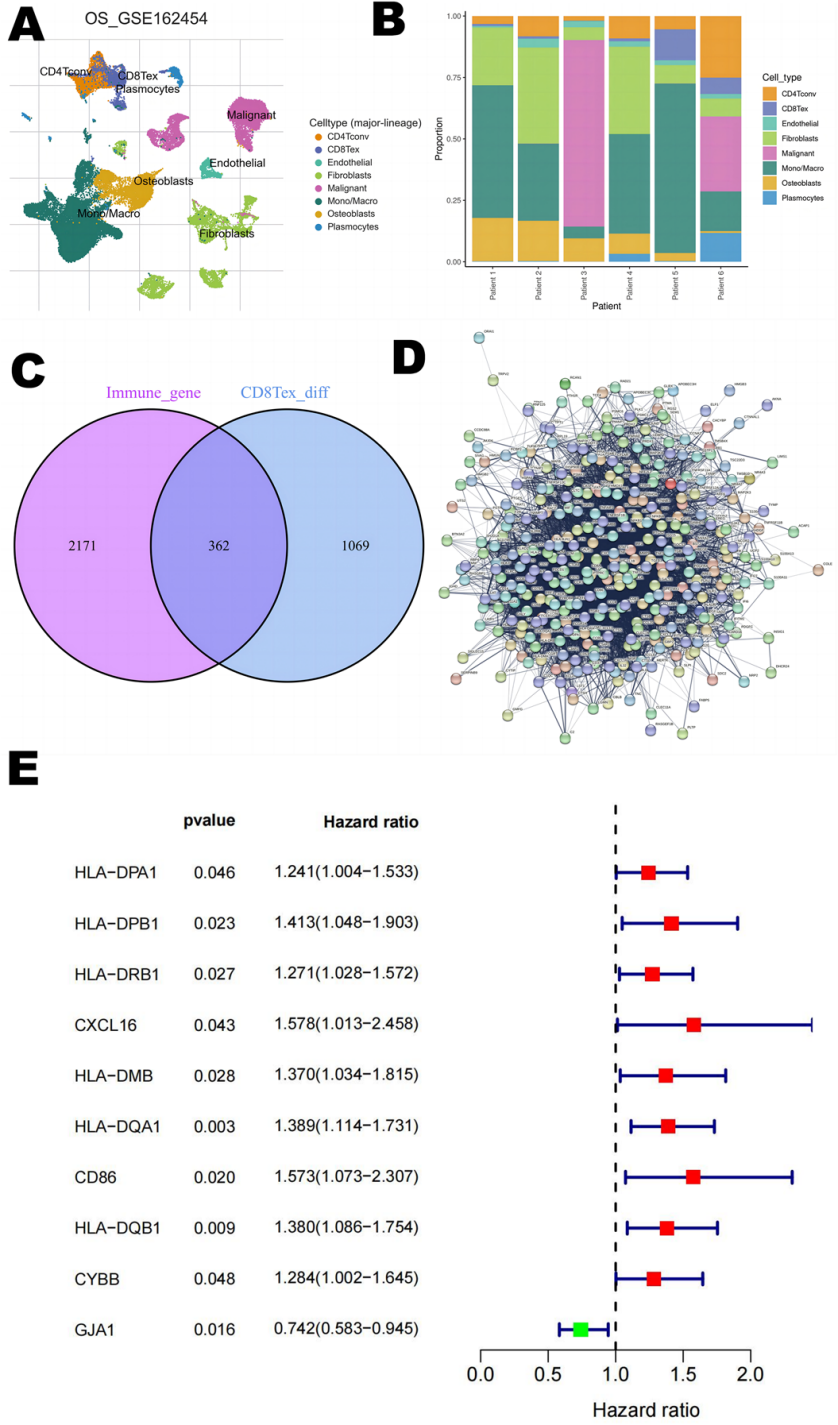
Construction of risk prognostic model (**A**,**B**) UMAP plot of single-cell sequencing in osteosarcoma patients and bar plots (**C**) T-cell differential genes and immune-related genes take intersections. (**D**) Protein–protein interactions interaction network of CD8-Tex-related genes. (**E**) Univariate Cox regression analysis obtained 109 candidate prognostic TRGs for OS.

**Figure 2:**
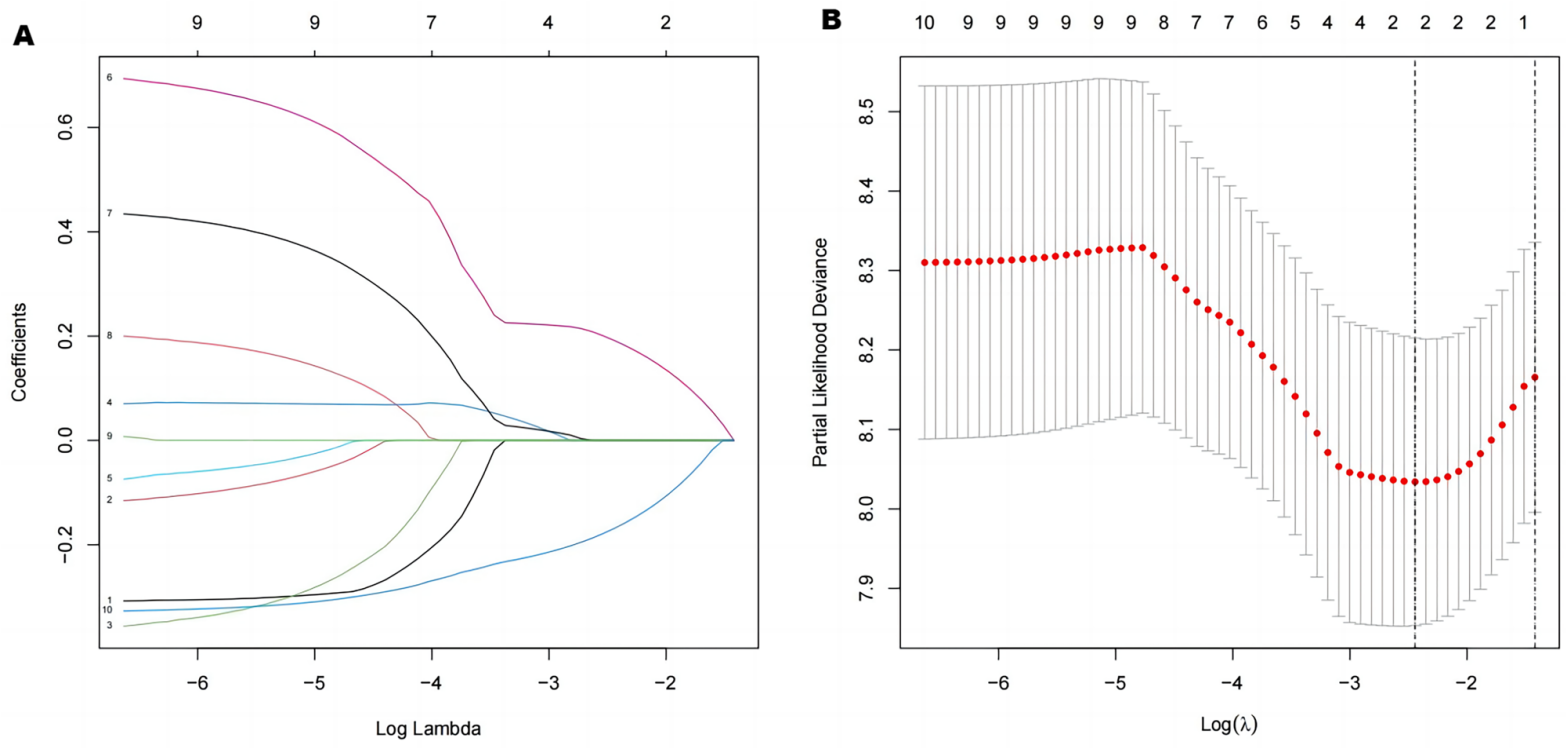
(**A**) LASSO regression analysis. (**B**) Selection of the optimal penalty parameter for LASSO regression.

As shown in Fig. 1, we searched the single-cell RNA sequencing data of osteosarcoma in the TISCH database (GSE162454) and extracted differential genes for the CD8Tex cell population. Intersections were taken with immune-related genes extracted from the database, and 362 intersecting genes were obtained. By univariate Cox analysis, 10 genes associated with CD8Tex cells were considered as potential prognostic indicators (genes were selected based on a p-value threshold of < 0.05) (Fig. 1). To reduce the risk of overfitting, least absolute shrinkage and selection operator (LASSO) Cox regression was subsequently performed. After applying the LASSO algorithm, a multivariate Cox regression model was used to identify the final gene set, which consisted of two robust genes (GJA1 and HLA-DQA1) that formed a prognostic signature for overall survival (Fig. 2).

### Correlation between TRS and prognosis of OS patients

To investigate the relationship between TRS and the prognosis of patients with osteosarcoma, we extracted clinical data of patients from the TARGET-OS database and the GEO database (GSE21257), respectively. The coefficients of two T-cell-related genes were used to determine the score for each patient. Gap junction alpha 1 (GJA1) gene is located in 6q22.31, encoding a member of cell junction protein family (Connexin 43), Zhang et al. showed that Connexin 43 inhibits osteosarcoma cell proliferation by mediating cell gap junction communication^21,22^. Comparatively, human leukocyte antigen (HLA)-DQA1 has been shown to be strongly associated with the onset and progression of several cancers^23,24^. In the LASSO Cox regression model, after selecting the best lambda values through cross-validation, the model gives the genes and their corresponding coefficients that have the most significant degree of influence on the survival data. The model formula shows the relationship between these genes and the survival data, describing how the survival risk can be predicted based on the gene expression, and the risk score is a numerical value calculated according to this formula, indicating the survival risk of an individual at a given moment. The risk score was calculated as follows: risk score = (− 0.169 × GJA1 expression) + (0.192 × HLA-DQA1 expression). Subsequently, participants were assigned to either the low-risk or high-risk group based on the median risk score. In both datasets, we were able to find that survival was lower in the high-risk group than in the low-risk group (p = 0.001, in the high-risk group, median survival time = 4.43 with 95% confidence interval (4.12, 4.73). For the low-risk group, median survival time = 6.18 with 95% confidence interval (4.94, 7.42), at the same time also we found similar trends in the validation set.

**Figure 3:**
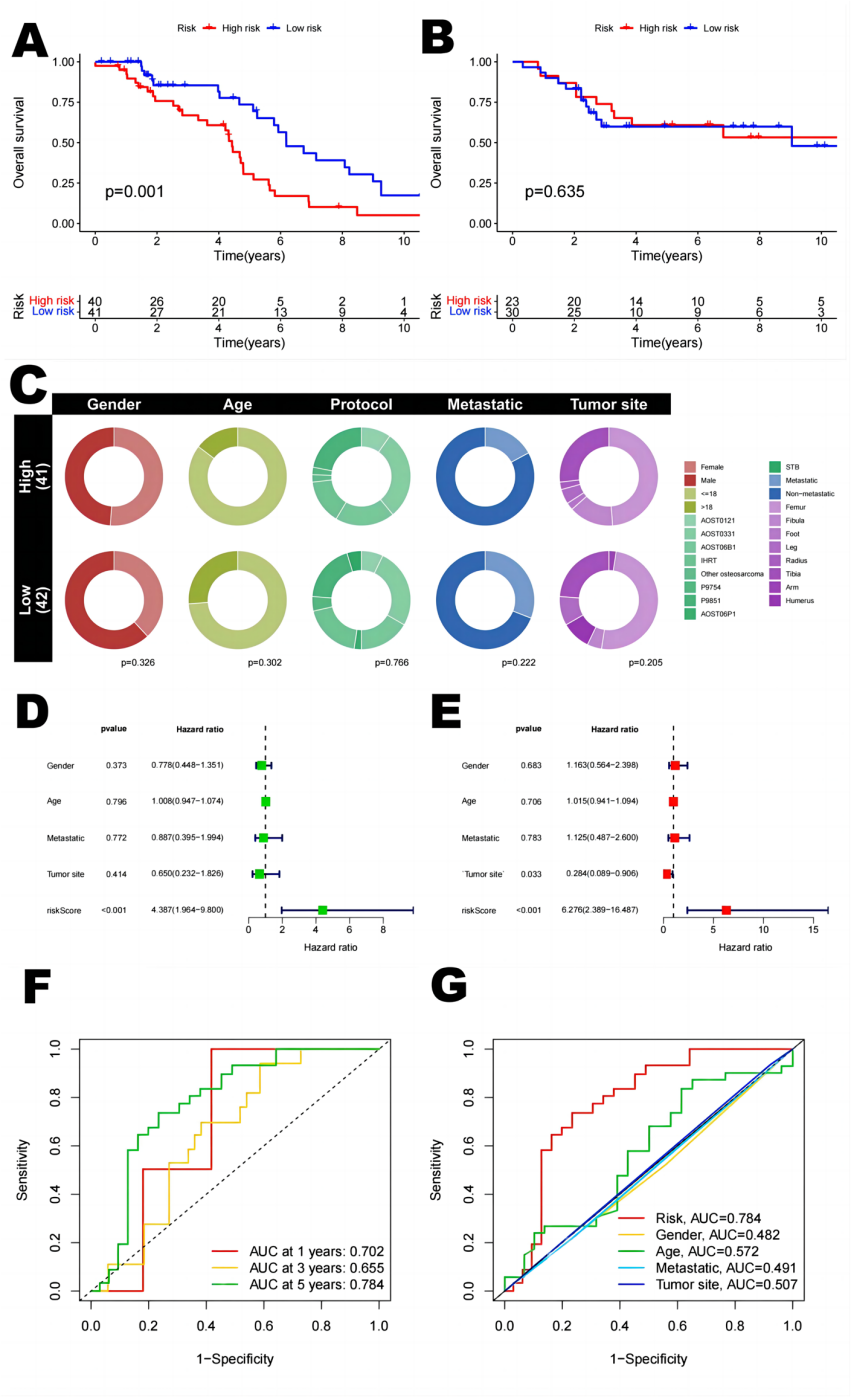
Kaplan–Meier survival analysis in OS patients and prognostic value of TRS. (**A**) Kaplan–Meier analysis of the overall survival of TARGET-OS set. (**B**) Kaplan–Meier analysis of the GEO set. (**C**) Clinical relevance circle chart. (**D**) Forest plot for univariate Cox regression analysis. (**E**) Forest plot for multivariate Cox regression analysis. (**F**) ROC curve and AUC at 1-year, 3-years and 5 years survival for TRS. (**G**) The ROC curve of the risk score and clinicopathological variables.

We used ROC curves to assess the predictive role of risk scores for 1, 3 and 5 year survival in patients with osteosarcoma. The AUC values for 1, 3 and 5 year survival were 0.702, 0.655, and 0.784, respectively (Fig. 3). The AUC for 5 year survival showed that the risk score (0.784) had a satisfactory predictive power (Fig. 3). Univariate and multivariate Cox regression analyses were used to evaluate the prognostic value of risk score and other factors. Risk score and metastasis were significant independent prognostic factors with HR values of 4.387 (95% CI 1.964–9.800, p < 0.001) for univariate analysis and 6.276 (95% CI 2.044–9.979, p < 0.001) for multivariate analysis (Fig. 3).

#### Functional analysis of high- and low-risk score groups

**Figure 4:**
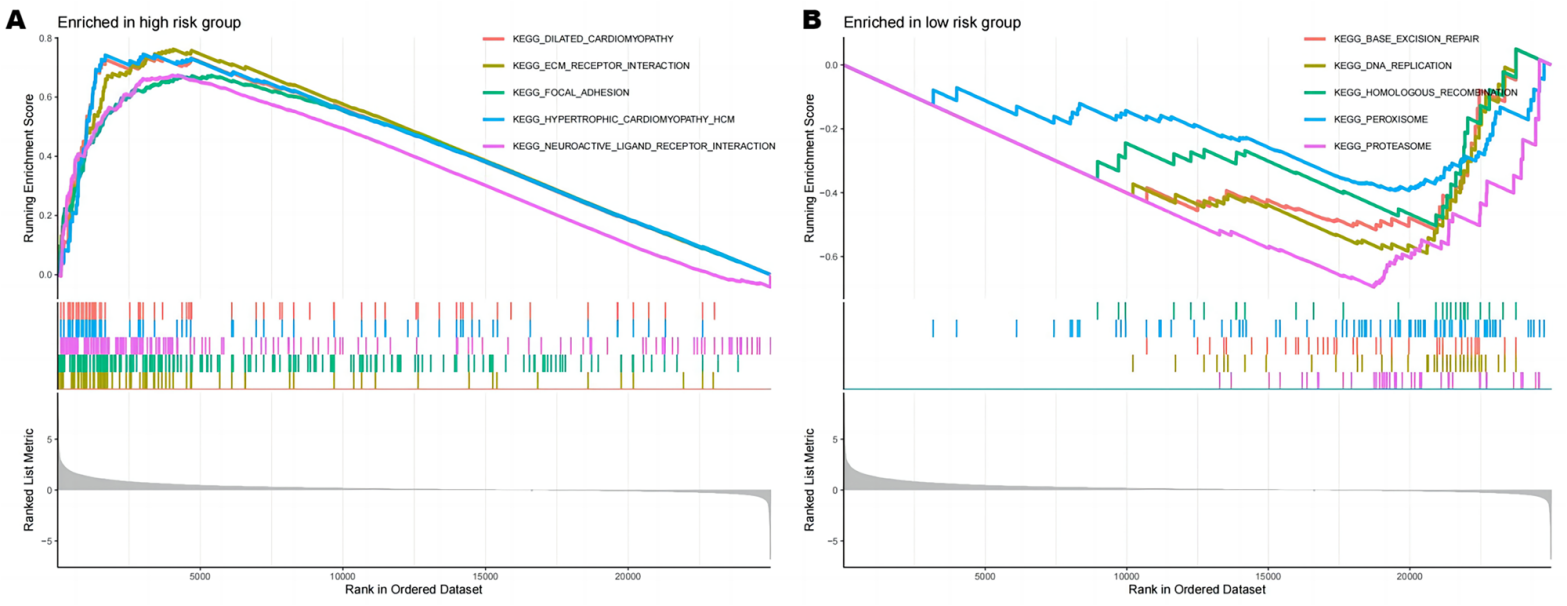
GSEA enrichment analysis.

A comprehensive analysis of the GSEA enrichment analysis methodology was performed to explore potential mechanisms leading to the different prognoses observed between the high- and low-risk groups. High risk was enriched for dilated cardiomyopathy, ECM receptor interaction, focal adhesion, hypertrophic cardiomyopathy (HCM), neuroactive ligand receptor interaction pathway. The low-risk group, on the other hand, was enriched in the base excision repair, DNA replication, homologous recombination, and peroxisome pathway (Fig. 4).

### Immune-related analysis of high- and low-risk score groups

**Figure 5:**
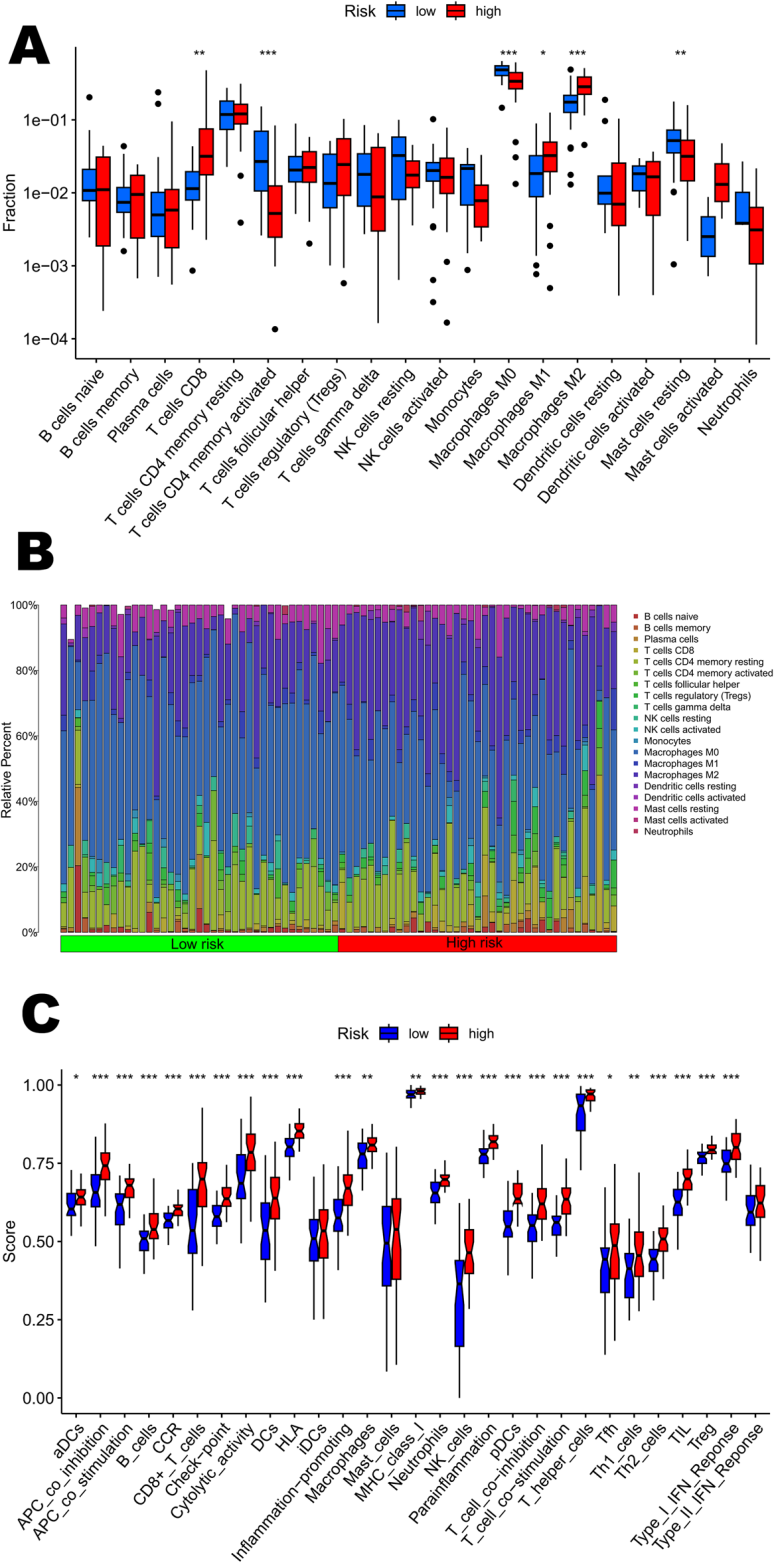
Immune related analysis in high- and low-risk groups. (**A**,**B**) Differences in the infiltration of immune cells between the high- and low- risk groups. (**C**) The correlation between the signature and immune functions.

To further investigate the relationship between risk scores and immune cell infiltration of tumors, the CIBERSORT algorithm was used to compare the proportions of 22 different types of immune cells between groups of low-risk or high-risk individuals. The results of the study showed that the low-risk group had higher proportions of M0 macrophages, and Mast cells resting (p < 0.05), whereas the high-risk group had higher proportions of T cells regulatory (Tregs), Macrophages M1, Macrophages M2, and Neutrophils ratio was higher, which correlated with immunosuppressive activity (p < 0.05) (Fig. 5). On this basis, the researcher employed the ssGSEA algorithm to further analyze the immune function. Figure 5 presents the results of the visualization of the significant differences in immune function between the two groups. ssGSEA algorithm was employed to provide a more comprehensive and in-depth understanding of the differences in immune function. The algorithm allowed us to delve into the functional activity of different immune cell types in both groups. These findings suggest that the low-risk group had lower levels of functional immune activity. In the low-risk group, the increase in CD4 natural T cells and M0 macrophages could imply stronger immune responses and cell-mediated immune protection. In contrast, in the high-risk group, the increase in Tregs, macrophage M1 and M2 may reflect enhanced immune regulation as well as increased inflammatory response, which may be associated with disease development and progression, providing important clues for future research and treatment. We then investigated the potential association between risk scores and immune checkpoint gene expression levels. The expression levels of immune checkpoint genes belonging to the Siglec family, such as SIGLEC7, SIGLEC8, SIGLEC9, SIGLEC10, SIGLEC11, SIGLEC14, and SIGLEC16, were significantly elevated in patients in the high-risk group (Supplementary Figure).

## Discussion

Osteosarcoma is a malignant swelling of bone tissue that occurs primarily in the adolescent and young adult population. Commonly used treatments include neoadjuvant chemotherapy, surgery, and adjuvant chemotherapy^25^. In the present study, the characterization of CD8 Tex cells was generated and subsequently evaluated for its correlation with overall survival in patients with osteosarcoma. Gene enrichment analysis was performed to explore the underlying mechanisms involved. The present study is the first to explore the genetic signature associated with CD8 Tex cells in osteosarcoma and marks an important step in our understanding of the immune environment of this malignancy.

The integration of the identified gene signature into clinical practice was achieved by establishing a risk score based on the expression of two T-cell-related genes (GJA1 and HLA-DQA1). GJA1 is the major protein expressed in epithelial tissues. Previous studies have shown that GJA1 expresses excellent cancer-inhibitory effects in a variety of cancers, which is consistent with our findings^26,27^. Although there are no definitive studies supporting a clear link between HLA-DQA1 and osteosarcoma, several studies seem to reveal a positive correlation with certain malignancies^28,29^. CD8 Tex cells are a special type of CD8+ T cells that play an important role in anti-tumor immunity.GJA1 encodes the Connexin 43 protein, which plays a key role in intercellular communication and may influence the interaction between tumor cells and immune cells, thereby affecting immune responses and tumor growth. Meanwhile, HLA-DQA1 is one of the MHC-class-II molecules involved in antigen presentation and immune regulation, and its variant or expression level may affect tumor recognition and attack by CD8Tex cells. In the biological mechanism of osteosarcoma, immune response regulation plays a key role. The activity of CD8Tex cells directly affects the growth and spread of tumor cells, while the expression of GJA1 and HLA-DQA1 may regulate the strength and direction of immune response. In addition, interactions in the tumor microenvironment also have an important impact on tumor progression and therapeutic response. The Connexin 43 protein encoded by the GJA1 gene may regulate signaling between tumor cells and immune cells, affecting the infiltration and activity of immune cells, which in turn affects the prognosis of the patient. Variants of HLA-DQA1 may lead to an increase in immune tolerance, which may cause tumor cells to escape from immunosurveillance that exacerbates disease progression. The association between TRS and patient prognosis was investigated using clinical data from the TARGET-OS and GEO databases. The results revealed a significant difference in survival between the low-risk and high-risk groups, emphasizing the potential clinical relevance of the identified gene signature. The calculated risk score demonstrated satisfactory predictive power, as evidenced by the AUC values for 1, 3, and 5 year survival. Univariate and multivariate Cox regression analyses further underscored the independent prognostic value of TRS, positioning it as a promising tool for risk stratification in osteosarcoma patients.

The functional analysis of the high- and low-risk score groups provided insights into the underlying biological processes associated with the identified gene signature. Enrichment analysis revealed distinct pathways enriched in each group, shedding light on potential mechanisms influencing prognosis. Notably, the high-risk group exhibited enrichment in pathways related to cardiac conditions, focal adhesion, and neuroactive ligand-receptor interaction, while the low-risk group showed enrichment in DNA repair and replication pathways. This suggests a potential connection between the immune status reflected in the gene signature and broader cellular processes that may influence the differential prognosis of patients with osteosarcoma.

The immune-related analysis further delved into the TME by employing the CIBERSORT algorithm to assess immune cell infiltration. The results highlighted significant differences in the composition of immune cells between low- and high-risk groups. Notably, the low-risk group demonstrated higher proportions of M0 macrophages, and resting Mast cells, indicating a more active immune response. In contrast, the high-risk group exhibited higher proportions of immunosuppressive cell types, including Tregs and both M1 and M2 macrophages, aligning with the notion of an immunosuppressive TME in high-risk osteosarcoma patients. Tregs are an important component of TME and are strongly associated with the prognosis of patients with osteosarcoma^30^. Macrophages, on the other hand, appear to play an important role in the pathogenesis of osteosarcoma. Within the tumor microenvironment, tumor-associated macrophages (TAMs) take center stage as the predominant infiltrating immune cells, characterized by their ability to undergo phenotypic polarization. Initially, TAMs predominantly adopt an M1 proinflammatory phenotype in the early stages of tumorigenesis, instigating an immune response that hampers tumor progression^31^. However, as the tumor advances, TAMs undergo a gradual shift toward an M2 functional phenotype^32^. This transition enhances their involvement in tumor angiogenesis and immunosuppression, fostering an environment conducive to tumor growth. Furthermore, considering the importance of RNA modification in immune regulation, Zhang et al. conducted a study on the involvement of m6A regulator-mediated RNA methylation modification patterns in the regulation of the immune microenvironment in periodontitis, revealing the role of RNA methylation in the immune microenvironment^33^. This study provides an interesting perspective on the possible link between RNA methylation and immune response in osteosarcoma and offers new ideas for understanding the dynamics of the tumor microenvironment, and further studies may reveal the potential role of RNA modification in osteosarcoma immune regulation.

The ssGSEA algorithm provided additional insight into immune function, showing that the low-risk group exhibited higher levels of immune function activity. Moreover, the association between risk scores and immune checkpoint gene expression levels emphasized the immunosuppressive nature of the high-risk group. The Siglec family functions as checkpoints in the immune cell response in human diseases such as cancer and autoimmune disorders, hence their interest as targets for therapeutic intervention^34,35^. They have been studied for many years as therapeutic targets in cancers^36^, including myeloid leukemia (Siglec-6)^37^, pancreatic cancer (Siglec-7 and Siglec-9)^38^, and others. In recent years, the emerging role of Siglec-15 in bone biology and cancer has also been gradually explored^39^. However, unfortunately, in the present study, Siglec-15 was not significantly different in the two groups. In this study, we investigated the association between risk scores and immune checkpoint gene expression and found that the levels of Siglec family genes (SIGLEC7, SIGLEC8, SIGLEC9, SIGLEC10, SIGLEC11, SIGLEC14, and SIGLEC16) were significantly elevated in the high-risk group. Together, these results suggest that risk scores in osteosarcoma patients may be associated with different immune profiles, which may influence the efficacy of treatment strategies. The upregulation of immune checkpoint genes in the high-risk group implies possible immunosuppression, highlighting the importance of considering immune-related factors when developing therapies for osteosarcoma.

The findings of this study hold several implications for the future treatment of osteosarcoma. Firstly, the identified CD8 Tex cell-associated gene signature serves as a potential biomarker for risk stratification, aiding clinicians in predicting patient outcomes and guiding treatment decisions. The integration of this signature into routine clinical practice could enhance the precision of prognostic assessments, enabling more tailored and effective therapeutic strategies. Moreover, the study emphasizes the importance of considering the immune landscape in osteosarcoma, particularly the role of CD8 Tex cells. The identified genes associated with CD8 Tex cells provide potential targets for immunotherapeutic interventions. Strategies that modulate these genes or enhance the immune response may be explored to overcome the immunosuppressive TME observed in high-risk patients. The functional and immune-related analyses shed light on the molecular and cellular processes that underlie the observed differences in prognosis. This knowledge opens avenues for the development of targeted therapies that address not only the tumor cells but also the broader microenvironment, potentially improving treatment outcomes.

The current study has several limitations that should be acknowledged. Firstly, the prognostic model was developed and validated using a single retrospective data source, which may introduce inherent biases and limit the generalizability of the findings. Secondly, due to the small sample size, there may be a risk of sample bias, resulting in compromised generalizability of the findings. In addition, the retrospective collection of data may have resulted in incomplete or inaccurate information, thus limiting a comprehensive understanding of the genetic characteristics of CD8 Tex cells. Future studies should aim to validate these findings in larger prospective cohorts. By expanding the sample size and employing a prospective design, the prevalence of CD8 Tex cell gene characterization can be better captured and the clinical applicability of the findings can be improved. In addition, it is recommended that future studies focus on a more comprehensive assessment of the function and impact of CD8 Tex cells to further understand their role in the immune response. Lastly, the available database provided only limited clinical information, restricting the depth of our analysis. Therefore, to strengthen the validity and applicability of our prognostic signature, future research should involve a larger and more diverse cohort, preferably through a prospective study design. This would allow for a more comprehensive assessment of the predictive value of the signature across various clinical contexts and improve the overall reliability of our findings.

In summary, this study contributes valuable insights into the molecular and immune characteristics of osteosarcoma, paving the way for a more nuanced understanding of the disease and offering potential avenues for therapeutic advancements. The integration of the identified gene signature and risk score into clinical practice could significantly impact patient care by facilitating personalized treatment approaches and improving overall outcomes for osteosarcoma patients. Further validation and prospective studies are warranted to solidify the clinical applicability of these findings and to explore the full potential of the identified gene signature in shaping the future landscape of osteosarcoma treatment.

## Method

### Single cell analysis

CD8 Tex cells in osteosarcoma were analyzed at the single-cell subset level using data from the Tumor Immunology Single Cell Center (TISCH) at http://tisch.comp-genomics.org/, a comprehensive scRNA-seq database dedicated to the study of the tumor microenvironment. Notably, TISCH provides detailed single-cell level annotations for various cell types, facilitating the exploration of different cancer types^40^. Major cell types in this dataset include immune cells, stromal cells, and malignant cells. The data quality control process was analyzed using the Seurat package (version 3.1.1; https://satijalab.org/seurat/install.html)^41,42^. The single-cell data had a gene number < 300 and > 4500; those with a mitochondrial gene number of > 10% were considered to be low-quality cells, and these were directly filtered out. The Harmony package (version 1.0; https://github.com/immunogenomics/Harmony) was then used to eliminate the batch effect of the cellular data^43^. Primary cell cluster analysis was performed using the FindClusters function of the Seurat package (resolution = 0.15), and the visual clustering results were presented through performing uniform manifold approximation and projection (UMAP) dimension reduction analysis.

### Construction of PPI network

The search tool for the retrieval of interacting genes (STRING) is an online tool that assesses protein–protein interaction (PPI) network information^44^. STRING (version 10.5) was used to evaluate the potential PPI relationships among those DEGs.

### Data collection and processing

Publicly available datasets were analyzed in this study. This data can be found here: the Tumor Immunology Single Cell Center (TISCH) (http://tisch.comp-genomics.org/), The Cancer Genome Atlas (https://portal.gdc.cancer.gov/), and the Gene Expression Omnibus (GEO) database (https://www.ncbi.nlm.nih.gov/geo/) for the GSE21257 dataset. The mRNA expression data and clinical details for osteosarcoma patients (TARGET-OS dataset) were obtained from the Cancer Genome Atlas Program (TCGA) database (https://portal.gdc.cancer.gov/). The GSE21257 dataset from the Gene Expression Omnibus database supplied as an external validation set mRNA expression data and clinical information for osteosarcoma specimens.

### Development of prognostic genes signature

A training set of 84 samples consisting of survival and expression data from the TARGET-OS dataset was used as the training set. The GSE21257 dataset provided 53 samples for an external validation cohort. Univariate Cox analysis was used as an initial screen for CD8Tex cell-associated genes associated with prognosis (selection of genes was based on a p-value threshold of < 0.05). To ensure convergence to an optimal solution during training, we chose to perform 1000 iterations in the LASSO algorithm to ensure robust gene selection. Using the LASSO Cox regression model to select the optimal subset of prognostic genes, by iterating the LASSO algorithm 1000 times in the training set, we identified the optimal subset of genes that form the CD8 Tex cell gene signature (TRS).

Lasso Cox regression analysis involves first screening variables using Lasso regression and then constructing Cox regression models to analyze prognostic effects. Regression modeling was performed using the “glmnet”package^45^. The LASSO regression was used first for variable selection, and then the Cox regression model was constructed to analyze the prognostic effects. Lasso can realize variable selection while model parameter estimation, which can better solve the problem of multiple covariance in the regression analysis and explain the results well. The Lasso regression algorithm uses L1 paradigm for shrinkage penalization, and penalization correction is applied to the coefficients of variables that do not contribute much to the dependent variable, and the coefficients of variables that do not contribute much to the dependent variable will be penalized. The coefficients of the variables are penalized to correct the coefficients of some less important variables, and the coefficients of the important variables are kept greater than 0, in order to reduce the number of covariates in Cox regression. Compared with traditional survival analysis, the LASSO Cox regression model can effectively prevent the problem of multicollinearity between variables or the number of variables is larger than the sample size^46^.

LASSO Cox regression can be regarded as a stand-alone model that combines the properties of LASSO regression and Cox regression. Instead of just applying LASSO to the effects of univariate Cox regression outputs, it simultaneously performs variable selection and regression coefficient estimation by means of L1-paradigm regularization to optimize the performance of the model.

Subsequently, risk scores were computed using the linear combination of each selected gene, employing the formula: Risk score = ∑ (coef (β) * EXP(β)), where β signifies the regression coefficient. Patients were stratified into high- and low-risk groups based on the median risk score as the threshold. Kaplan–Meier survival analysis was employed to explore clinical differences between these groups. Model performance was assessed through ROC curve analysis and C-index calculations. Additionally, stratified analysis was conducted to evaluate the additional prognostic value of the TRS.

### Construction of nomogram

A predictive nomogram was developed to estimate the 1-, 3-, and 5 year survival rates for osteosarcoma (OS) patients, incorporating the risk score derived from the CD8 Tex cell gene signature and relevant clinicopathological factors such as age, gender, race, and metastasis. The accuracy of the nomogram’s predictions was subsequently assessed through the construction of a calibration curve, comparing the actual overall survival with the predicted survival rates.

### Functional enrichment analysis in the TARGET-OS cohort

The cohort was stratified into high- and low-risk groups based on the predefined risk score threshold. Following this partitioning, gene expression fold changes were examined using the “limma” R package. Subsequently, pathway analysis was conducted with the “clusterProfiler” R package, focusing on the identification of significantly enriched pathways within the reference gene set for both the high- and low-risk groups. The reference gene set was specifically defined as the hallmark gene sets described by Subramanian et al^47^.

### Immune-related analysis of TRS

To delve into the immune-related implications of the developed TRS (CD8 Tex cell gene signature), a comprehensive analysis was conducted. The single-sample gene set enrichment analysis (ssGSEA) algorithm, implemented through R packages such as limma, GSVA, and GSEABase, was employed to assess the differences in immune function between the high- and low-risk groups as defined by TRS^48^. The TME and immune cell infiltration were further evaluated using the ESTIMATE and CIBERSORT algorithms, respectively, allowing for a nuanced understanding of the proportions of their components^49,50^. Additionally, the study delved into the association between the expression levels of immune checkpoint genes and the two risk-defined groups.

### Supplementary Information

Supplementary Figure 1.
