# Supplementary material for: Importance of CD8 Tex cell-associated gene signatures in the prognosis and immunology of osteosarcoma
Source: Sci Rep. 2024 Apr 29;14:9769. doi: 10.1038/s41598-024-60539-z (PMC11058769; doi:10.1038/s41598-024-60539-z)

**Supplementary Figure.** Analysis of differences in immune checkpoints between high- and low-risk groups (Siglec family)


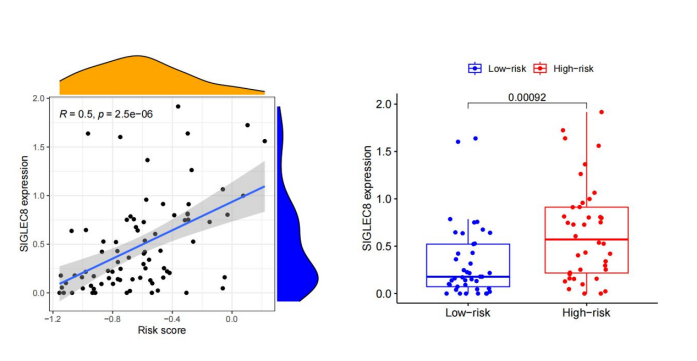

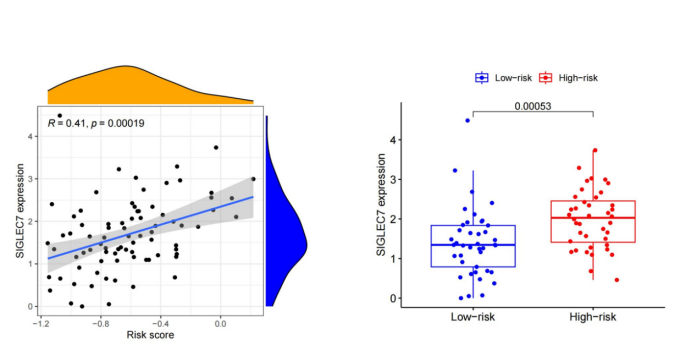

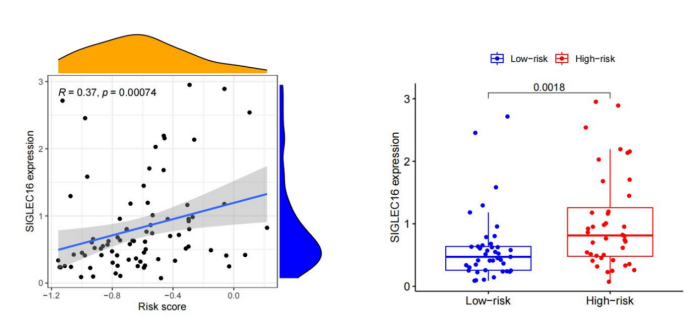

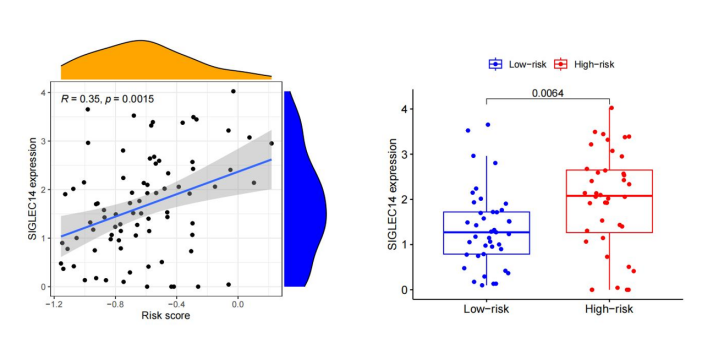

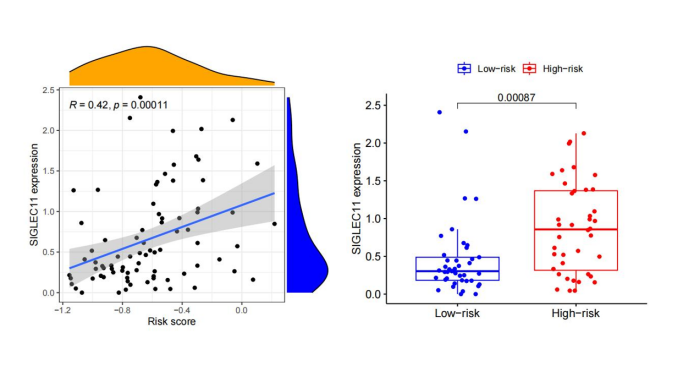


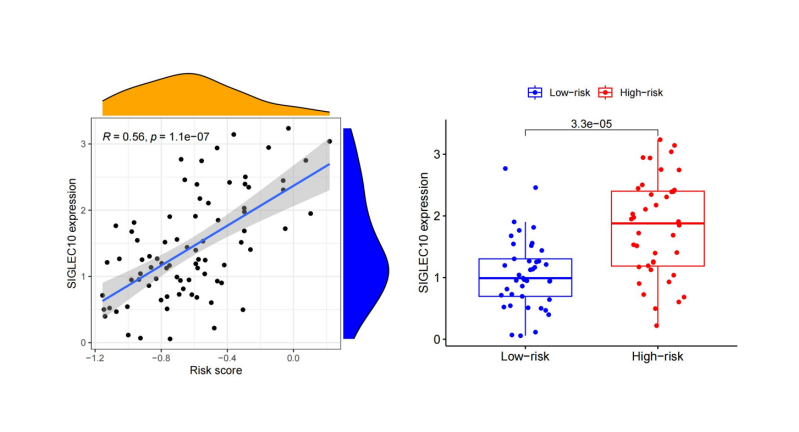

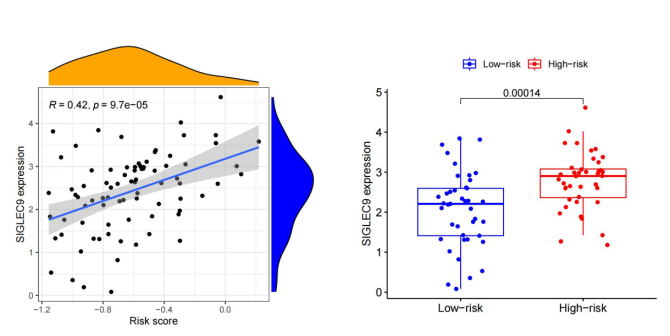

Supplement: Supplementary file 1 — Supplementary Figure 1. [file 41598_2024_60539_MOESM1_ESM.docx]
